# Variation in haemodynamic monitoring for major surgery in European nations: secondary analysis of the EuSOS dataset

**DOI:** 10.1186/s13741-015-0018-8

**Published:** 2015-09-23

**Authors:** Tahania Ahmad, Christian M. Beilstein, Cesar Aldecoa, Rui P. Moreno, Zsolt Molnár, Vesna Novak-Jankovic, Christoph K. Hofer, Michael Sander, Andrew Rhodes, Rupert M. Pearse

**Affiliations:** Barts and The London School of Medicine and Dentistry, Queen Mary University of London, London, UK; Hospital Universitario Rio Hortega, Valladolid, Spain; Unidade de Cuidados Intensivos Neurocríticos, Centro Hospitalar de Lisboa Central, Lisbon, Portugal; University of Szeged, Szeged, Hungary; Ljubljana University Medical Centre, Ljubljana, Slovenia; Triemli City Hospital of Zurich, Zurich, Switzerland; Charité University, Berlin, Germany; St. George’s University of London, London, UK; Adult Critical Care Unit, Royal London Hospital, London, E1 1BB UK

**Keywords:** Major surgery, Perioperative medicine, Cardiac output monitoring, Haemodynamic monitoring

## Abstract

**Background:**

The use of cardiac output monitoring may improve patient outcomes after major surgery. However, little is known about the use of this technology across nations.

**Methods:**

This is a secondary analysis of a previously published observational study. Patients aged 16 years and over undergoing major non-cardiac surgery in a 7-day period in April 2011 were included into this analysis. The objective is to describe prevalence and type of cardiac output monitoring used in major surgery in Europe.

**Results:**

Included in the analysis were 12,170 patients from the surgical services of 426 hospitals in 28 European nations. One thousand four hundred and sixteen patients (11.6 %) were exposed to cardiac output monitoring, and 2343 patients (19.3 %) received a central venous catheter. Patients with higher American Society of Anesthesiologists (ASA) scores were more frequently exposed to cardiac output monitoring (ASA I and II, 643 patients [8.6 %]; ASA III–V, 768 patients [16.2 %]; *p* < 0.01) and central venous catheter (ASA I and II, 874 patients [11.8 %]; ASA III–V, 1463 patients [30.9 %]; *p* < 0.01). In elective surgery, 990 patients (10.8 %) were exposed to cardiac output monitoring, in urgent surgery 252 patients (11.7 %) and in emergency surgery 173 patients (19.8 %). A central venous catheter was used in 1514 patients (16.6 %) undergoing elective, in 480 patients (22.2 %) undergoing urgent and in 349 patients (39.9 %) undergoing emergency surgery. Nine hundred sixty patients (7.9 %) were monitored using arterial waveform analysis, 238 patients (2.0 %) using oesophageal Doppler ultrasound, 55 patients (0.5 %) using a pulmonary artery catheter and 44 patients (2.0 %) using other technologies. Across nations, cardiac output monitoring use varied from 0.0 % (0/249 patients) to 27.5 % (19/69 patients), whilst central venous catheter use varied from 5.6 % (7/125 patients) to 43.2 % (16/37 patients).

**Conclusions:**

One in ten patients undergoing major surgery is exposed to cardiac output monitoring whilst one in five receives a central venous catheter. The use of both technologies varies widely across Europe.

**Trial registration:**

ClinicalTrials.gov Identifier: NCT01203605. Date of registration: 15.09.2010.

**Electronic supplementary material:**

The online version of this article (doi:10.1186/s13741-015-0018-8) contains supplementary material, which is available to authorized users.

## Background

More than 312 million surgical procedures are performed worldwide each year with an estimated mortality between 1 and 4 % [1, 2]. Postoperative complications and death are most frequent amongst high-risk patients, who are older, have co-morbid disease and undergo major surgery [3]. The dose of intravenous fluid and vasoactive drugs has an important effect on patient outcomes following major gastrointestinal surgery [4]. These treatments are prescribed according to subjective criteria leading to wide variation in clinical practice [5–9]. One potential solution to this problem is the use of cardiac output monitoring to guide administration of intravenous fluid and vasoactive drugs [4].

The use of perioperative cardiac output monitoring remains controversial, with differing interpretations of the evidence base for this treatment approach [10–18]. Recent evidence from a large clinical trial suggests that the benefit associated with this technology may be more marginal than previously believed, whilst some commentators have raised safety concerns [4, 19, 20]. Cardiac output monitoring has been recommended for patients undergoing selected types of major surgery both by the National Institute for Health and Care Excellence (NICE) in the UK and in a report commissioned by the Centers for Medicare and Medicaid Services in the USA [21, 22]. However, clinician surveys and anecdotal evidence suggest there is wide variation in the use of this technology [6–9].

Across Europe, little is known about the use of cardiac output monitoring in non-cardiac surgery. A previously published 7-day cohort study described mortality and perioperative care in 28 European nations (European Surgical Outcomes Study [EuSOS]) [2]. Data were collected which describe the use of cardiac output monitoring and central venous catheterisation. We performed a secondary analysis of the EuSOS dataset to describe the prevalence and types of cardiac output monitoring in patients undergoing major surgery across European nations.

## Methods

### Ethics

Ethics requirements differed by country. In Denmark, centres were exempt from ethics approval as the study was deemed to be a clinical audit. In all other nations, formal ethics approval was obtained. For the United Kingdom, approval was given by the Oxfordshire Research Ethics Committee B (Harrow, United Kingdom, 15th November 2010, chair Prof. M. Rees, reference number 10/H0605/72). In Finland, informed consent was obtained for all participants as required by the ethics committee.

### Setting

This is a secondary analysis of the previously published European Surgical Outcomes Study, a 7-day observational cohort study including consecutive patients undergoing inpatient non-cardiac surgery in April 2011 [2].

### Data collection

Eligible for EuSOS were consenting patients aged 16 years and over undergoing inpatient non-cardiac surgery, irrespective of the chosen anaesthetic technique. We excluded patients undergoing planned day-case surgery, cardiac surgery, neurosurgery, radiological or obstetric procedures. From this dataset, we only included patients undergoing major surgery (duration more than 90 min as defined in the EuSOS protocol) in this analysis. Use of haemodynamic monitoring in the operating room was recorded on a paper case record form in the following categories: central venous catheter, arterial waveform analysis (including both calibrated and uncalibrated analysis), oesophageal Doppler ultrasound, pulmonary artery catheter and other. Data were assessed for completeness and checked for plausibility and consistency. A list of participating hospitals and full details of the methodology of the study can be found in the original publication [2].

### Statistical analysis

A descriptive analysis was carried out according to a prospectively written statistical analysis plan. Data are presented as mean (standard deviation) for continuous variables and absolute or relative frequencies as percentages for categorical variables. Baseline characteristics of patient groups were tabulated and differences between groups compared using Pearson’s chi-square (*χ*^2^) test or *t*-test as appropriate. Prevalence of each type of haemodynamic monitoring was assessed per urgency of surgery, surgical speciality and per nation. Countries with less than ten recruited patients were excluded from graphs presenting per nation data. All analysis was carried out using STATA MP 13.1 (STATA Corp, USA).

## Results

### Baseline characteristics

From the original EuSOS dataset (46,539 patients), we excluded 34,349 patients who did not undergo major surgery and 20 patients for missing haemodynamic monitoring data. In total, 12,170 patients who underwent major surgery in 426 hospitals from 28 countries were included into this analysis. The patient flow diagram is available as an additional online file (Additional file 1: Figure S1). Baseline characteristics of patients are presented in Table 1 according to exposure to cardiac output monitoring and central venous catheter (CVC).

**Table 1:** Baseline characteristics of patients without/with cardiac output monitoring and with central venous catheter

	All	Without cardiac output monitoring	With cardiac output monitoring	p*	With central venous catheter
Age				<0.01	
Mean (SD)	61.7 (16.5)	61.4 (16.6)	63.6 (15.2)		62.9 (15.0)
Gender				<0.01	
Male	6290 (51.7 %)	5706 (90.7 %)	584 (9.3 %)		935 (14.9 %)
Female	5878 (48.3 %)	5048 (85.9 %)	830 (14.1 %)		1407 (23.9 %)
Current smoker	2329 (19.3 %)	2041 (19.1 %)	288 (20.5 %)	0.20	527 (22.7 %)
ASA Score				<0.01	
I and II	7400 (60.8 %)	6793 (91.4 %)	643 (8.6 %)		874 (11.8 %)
III, IV and V	4736 (39.0 %)	3978 (84.0 %)	768 (16.2 %)		1463 (30.9 %)
Urgency of surgery				<0.01	
Elective	9132 (75.1 %)	8142 (89.2 %)	990 (10.8 %)		1514 (16.6 %)
Urgent	2161 (17.8 %)	1909 (88.3 %)	252 (11.7 %)		480 (22.2 %)
Emergency	875 (7.2 %)	702 (80.2 %)	173 (19.8 %)		349 (39.9 %)
Surgical speciality				<0.01	
Orthopaedic	3984 (32.8 %)	3800 (95.4 %)	184 (4.6 %)		175 (4.4 %)
Breast	350 (2.9 %)	331 (94.6 %)	19 (5.4 %)		32 (9.2 %)
Gynaecology	1051 (8.7 %)	1005 (95.6 %)	46 (4.4 %)		70 (6.7 %)
Vascular	807 (6.7 %)	633 (78.4 %)	174 (21.6 %)		267 (33.3 %)
Gastrointestinal	2717 (22.3 %)	2144 (78.9 %)	573 (21.1 %)		971 (35.7 %)
Hepato-biliary	510 (4.2 %)	370 (72.6 %)	140 (27.5 %)		299 (58.6 %)
Plastic or cutaneous	192 (1.6 %)	188 (97.9 %)	4 (2.1 %)		22 (11.5 %)
Urology and kidney	1219 (10.0 %)	1073 (87.1 %)	146 (12.9 %)		266 (28.2 %)
Head and neck	684 (5.6 %)	638 (93.3 %)	46 (6.7 %)		64 (9.4 %)
Other	622 (5.1)	542 (87.1 %)	80 (12.9 %)		167 (26.9 %)
Co-morbid disorder					
No co-morbid disorder	6679 (54.9 %)	6095 (91.2 %)	584 (8.7 %)	<0.01	918 (13.7 %)
Cirrhosis	186 (1.5 %)	126 (67.7 %)	60 (32.3 %)	<0.01	97 (52.2 %)
Congestive heart failure	646 (5.3 %)	519 (80.3 %)	127 (19.7 %)	<0.01	196 (30.3 %)
COPD	1590 (13.1 %)	1352 (85.0 %)	238 (15.0 %)	<0.01	388 (24.4 %)
Coronary artery disease	1999 (16.5 %)	1685 (84.3 %)	314 (15.7 %)	<0.01	460 (23.1 %)
NIDDM	1119 (9.2 %)	973 (87.0 %)	146 (13.1 %)	0.12	245 (21.9 %)
Metastatic cancer	1014 (8.4 %)	815 (80.4 %)	199 (19.6 %)	<0.01	421 (41.5 %)
Stroke	719 (5.9 %)	601 (83.6 %)	118 (16.4 %)	<0.01	172 (23.9 %)
Hospital statistics					
Length of hospital stay (mean, SD)	8.9 (9.4)	8.5 (9.3)	12.4 (12.0)	<0.01	15.0 (12.9)
ICU admission	2534 (20.8 %)	1900 (17.7 %)	634 (44.8 %)	<0.01	1445 (61.7 %)
Mortality	667 (5.5 %)	535 (5.0 %)	142 (10.0 %)	<0.01	287 (12.2 %)
Total	12 170	10,754 (88.4 %)	1416 (11.6 %)		2343 (19.3 %)

Data presented as mean (SD) or *n* (%)

*ASA* American Society of Anesthesiologists, *ICU* intensive care unit, *NIDDM* non-insulin-dependent diabetes mellitus, *COPD* chronic obstructive pulmonary disease

*p value describes comparison of the patient groups with and without cardiac output monitoring

### Use of haemodynamic monitoring

**Fig. 1:**
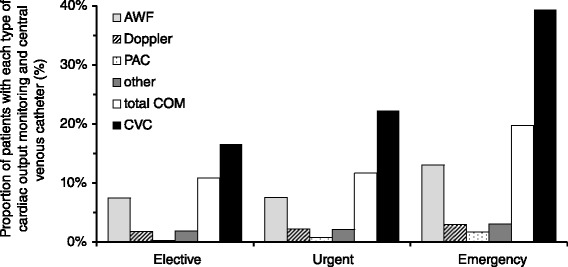
Use of cardiac output monitoring and central venous catheter per urgency of surgery. Data displayed as percentage per urgency of surgery. *AWF* arterial waveform analysis, *Doppler* Doppler ultrasound, *PAC* pulmonary artery catheter, *COM* cardiac output monitoring, *CVC* central venous catheter

**Fig. 2:**
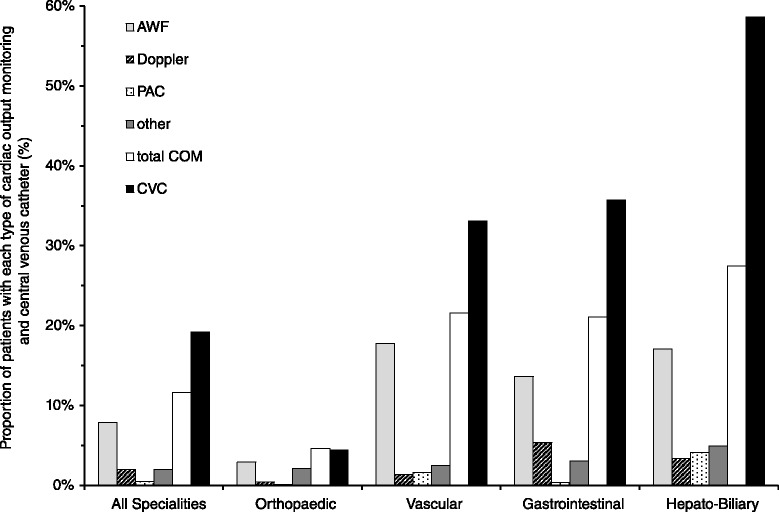
Use of cardiac output monitoring and central venous catheter overall and per surgical speciality. Data displayed as percentage overall/per surgical speciality. *AWF* arterial waveform analysis, *Doppler* Doppler ultrasound, *PAC* pulmonary artery catheter, *COM* cardiac output monitoring, *CVC* central venous catheter

**Fig. 3:**
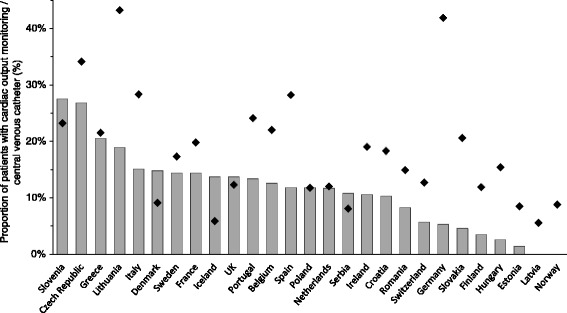
Variation in use of cardiac output monitoring and central venous catheter in European nations. Data is presented in % of patients with any type of cardiac output monitoring (*bar*) and central venous catheter (*black diamond*) per nation. Only nations with more than ten recruited patients are included into this graph. *UK* United Kingdom

One thousand four hundred and sixteen patients (11.6 %) were exposed to cardiac output monitoring, and 2343 (19.3 %) patients received a CVC. Six hundred eighty (5.6 %) patients received both cardiac output monitoring and a CVC. Patients exposed to cardiac output monitoring or central venous catheterisation had higher American Society of Anesthesiologists (ASA) scores, were slightly older and more likely to undergo urgent or emergency surgery (Table 1). Patients receiving cardiac output monitoring or a CVC had a longer hospital stay, were more likely to be admitted to intensive care and were more likely to die than patients who did not receive cardiac output monitoring (Table 1). Nine hundred sixty patients (7.9 %) were monitored using arterial waveform analysis, 238 (2.0 %) using oesophageal Doppler ultrasound, 55 patients (0.5 %) using a pulmonary artery catheter and 44 patients (2.0 %) using another technology. Arterial waveform analysis was the most frequently used technique across urgencies of surgery (Fig. 1), surgical specialities (Fig. 2) and nations. Country-level data describing the use of different monitoring techniques is available as an additional online file (see Additional file 1: Figure S2 and Table S1). Across European nations, cardiac output monitoring use varied from 0.0 % (0/249 patients) to 27.5 % (19/69 patients) and the use of CVC from 5.6 % (7/125 patients) to 43.2 % (16/37 patients) (Fig. 3).

## Discussion

The principal finding of this analysis was that one in ten patients undergoing major surgery is exposed to cardiac output monitoring and one in five patients receives a CVC. Cardiac output monitoring and CVC use were more frequent with increasing ASA score and increasing urgency of surgery. The use of both cardiac output monitoring and CVCs was associated with a longer hospital stay, more admissions to critical care and higher mortality. Importantly, there was wide variation in the use of cardiac output monitoring and CVCs across European nations. The most commonly used method of cardiac output monitoring was arterial waveform analysis.

There is very little published patient-level data describing the use of cardiac output monitoring either in individual countries or at an international level. However, questionnaire-based surveys amongst anaesthesiologists have been used to explore the use of cardiac output monitoring in high-risk surgical patients. The most widely cited of these is an online questionnaire survey conducted in 2011 by Cannesson et al. amongst members of the American Society of Anesthesiologists and the European Society of Anaesthesiology. From 368 respondents, 35 % routinely monitored cardiac output with similar numbers in Europe and in North America [6]. A similar survey of members of the Korean Society of Anesthesiologists revealed that 59 % of 139 respondents were using cardiac output monitoring [8]. The findings of a paper-based survey of Chinese anaesthetists showed that cardiac output monitoring was used by 13 % of 210 respondents [9]. In addition to the variation in use of cardiac output monitoring, these surveys also reveal differences in the monitoring technique used. This variability is supported by another online questionnaire-based survey, exploring the use of goal-directed therapy in major elective surgery amongst members of the Association of Anaesthetists of Great Britain and Ireland, the American Society of Anesthesiologists and the Australia and New Zealand College of Anaesthetists [7]. Overall, the findings of these clinician surveys are consistent with the findings of our analysis of patient-level data. The lower use of cardiac output monitoring in our data compared to the data provided by Cannesson et al. might be due to the broader inclusion criteria of our study [6]. Suggested explanations for the wide variation in clinical practice were local factors including availability of equipment, experience or education and national factors like reimbursement and guidelines [6, 21–23]. Involvement of clinicians in the development of, and therefore familiarity with, a certain device may also provide another explanation, which is why, for example, the use of PiCCO (PULSION Medical Systems, Germany) or LiDCO (LiDCO Ltd., United Kingdom) seems to be focused on their country of origin [6]. In addition, involvement of national opinion leaders in clinical trials improving the evidence base for certain devices might have contributed to the more extensive use of oesophageal Doppler monitoring in the United Kingdom compared to other nations [24–26]. Scepticism regarding possible benefits and harms is seen as another important contributor [6]. This in view of studies which suggested that fluid restriction might be equally beneficial as fluid optimization [19, 27, 28].

To our knowledge, this is the largest available patient dataset describing the use of haemodynamic monitoring across international boundaries. However, the study only provides evidence of activity in one 7-day period in participating centres and so may not provide an accurate reflection of activity over a longer time frame. The data were collected 4 years ago, and contemporary practice may have evolved since the original study. The small number of patients returned by a small number of hospitals in smaller countries may also have resulted in bias and a poor representation of patterns of monitoring use within those countries. In addition, the small patient numbers in some sub-categories limit the robustness of any analysis of variation between hospitals or potential clustering within hospitals, especially as some centres may specialise in particular types of surgery with consequent effects on care pathways and case mix. The study was not specifically designed for collection of data describing the use of haemodynamic monitoring and did not provide precise detail on the specific product used in each patient. No data were collected to describe how haemodynamic data were used, the target values for specific variables or which interventions were used to attain these targets. In addition, there is no data describing the indication for CVC placement. Due to the heterogeneity of cases and the limited sample size, we were not able to further analyse effects of haemodynamic monitoring on clinical outcomes.

## Conclusions

One in ten patients undergoing major surgery in Europe is exposed to cardiac output monitoring whilst one in five receives a central venous catheter. The use of both technologies varies widely across nations. Further research is needed to confirm the clinical value of cardiac output monitoring and how this technology might help to further reduce postoperative morbidity and mortality.

## Additional file

Additional file 1:
**Supplemental digital content.** Patient flow diagram (supplementary Figure 1), variation in the use of different types of cardiac output monitoring in European nations (supplementary Figure 2) and types of haemodynamic monitoring used in European nations (supplementary Table). (PDF 333 kb)

## Abbreviations

ASA: American Society of Anesthesiologists
AWF: arterial waveform analysis
COM: cardiac output monitoring
COPD: chronic obstructive pulmonary disease
CVC: central venous catheter
EuSOS: European Surgical Outcomes Study
ICU: intensive care unit
NICE: National Institute for Health and Care Excellence
NIDDM: non-insulin-dependent diabetes mellitus
PAC: pulmonary artery catheter
UK: United Kingdom
