# Supplementary material for: Variation in haemodynamic monitoring for major surgery in European nations: secondary analysis of the EuSOS dataset
Source: Perioper Med (Lond). 2015 Sep 23;4:8. doi: 10.1186/s13741-015-0018-8 (PMC4581514; doi:10.1186/s13741-015-0018-8)
Supplement: Additional file 1: — Supplemental digital content. Patient flow diagram (supplementary Figure 1), variation in the use of different types of cardiac output monitoring in European nations (supplementary Figure 2) and types of haemodynamic monitoring used in European nations (supplementary Table). (PDF 333 kb) [file 13741_2015_18_MOESM1_ESM.pdf]

Variation in haemodynamic monitoring for major surgery in European nations: secondary analysis of the EuSOS dataset

Supplemental Digital Content

***Tahania Ahmad, *Christian M. Beilstein, Cesar Aldecoa,**

Rui P. Moreno, Zsolt Molnár, Vesna Novak-Jankovic,

Christoph K. Hofer, Michael Sander, Andrew Rhodes and

Rupert M. Pearse

***authors made an equal contribution to the submitted work**

Supplementary Figure 1: Patient flow diagram

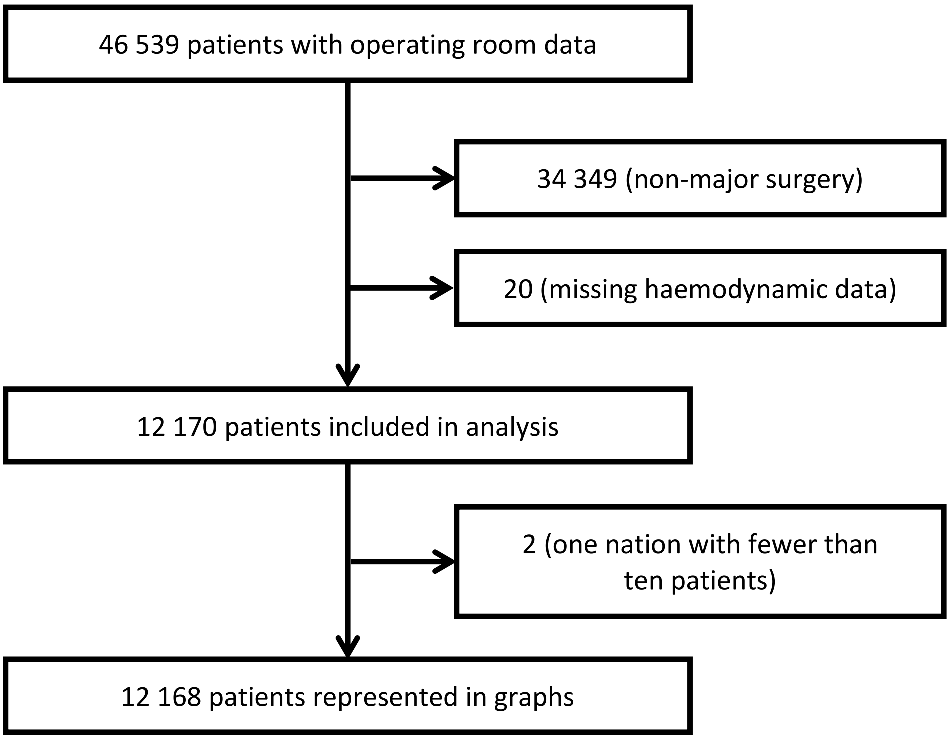

Supplementary Figure 2: Variation in use of different types of cardiac output monitoring in European nations

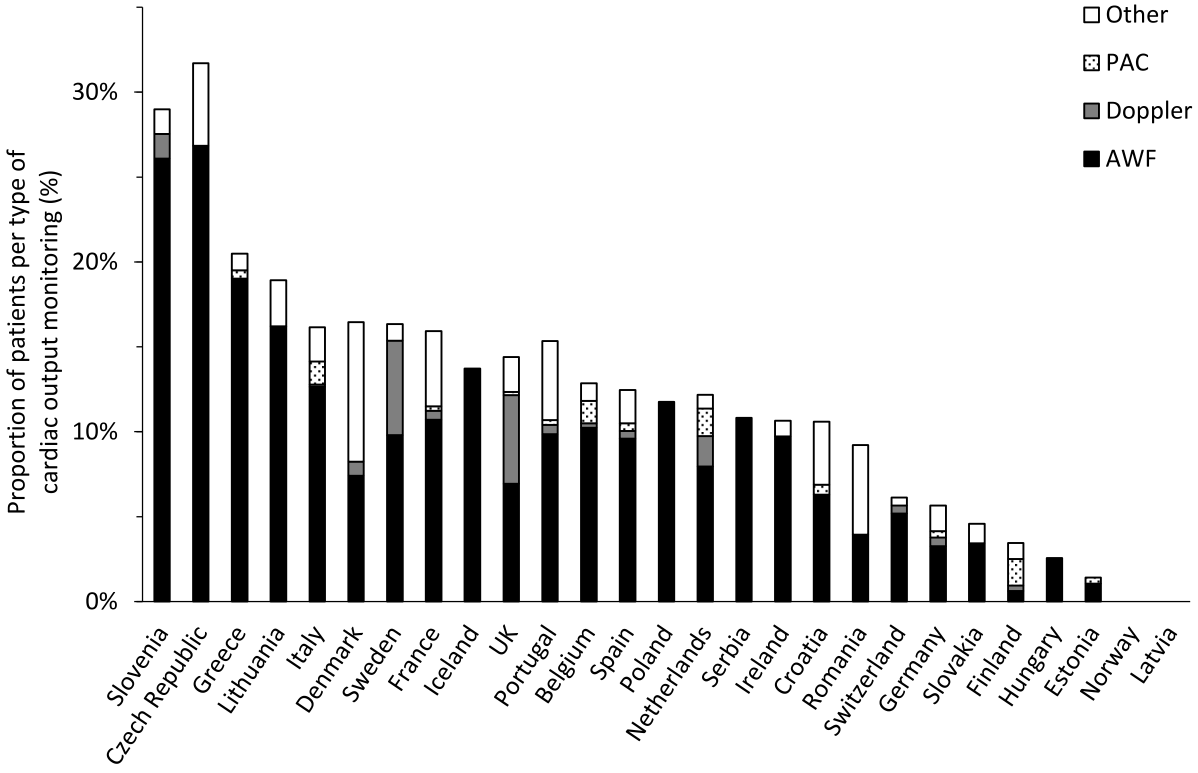

Data are presented in % per type of COM used per nation. Only nations with more than 10 recruited patients were included into this graph. Note that the use of multiple types of COM per patient was possible. PAC, pulmonary artery catheter; Doppler, Doppler ultrasound; AWF, arterial wave form analysis; UK, United Kingdom.

Supplementary Table: Types of haemodynamic monitoring used in European nations

Nation	N	AWF	Doppler	PAC	Other	Total COM	CVC
Belgium	381	39 (10.2)	1 (0.3)	5 (1.3)	4 (1.0)	48 (12.6)	84 (22.0)
Croatia	349	22 (6.3)	0 (0.0)	2 (0.6)	13 (3.7)	36 (10.3)	64 (18.3)
Cyprus	2	1 (50.0)	0 (0.0)	0 (0.0)	1 (50.0)	2 (100.0)	2 (100.0)
Czech Republic	41	11 (26.8)	0 (0.0)	0 (0.0)	2 (4.9)	11 (26.8)	14 (34.1)
Denmark	243	18 (7.4)	2 (0.8)	0 (0.0)	20 (8.2)	36 (14.8)	22 (9.1)
Estonia	283	3 (1.1)	0 (0.0)	1 (0.4)	0 (0.0)	4 (1.4)	24 (8.5)
Finland	318	2 (0.6)	1 (0.3)	5 (1.6)	3 (0.9)	11 (3.5)	38 (11.9)
France	383	41 (10.7)	2 (0.5)	1 (0.3)	17 (4.4)	55 (14.4)	76 (19.8)
Germany	797	26 (3.3)	4 (0.5)	3 (0.4)	12 (1.5)	42 (5.3)	333 (41.8)
Greece	405	77 (19.0)	0 (0.0)	2 (0.5)	4 (1.0)	83 (20.5)	87 (21.5)
Hungary	117	3 (2.6)	0 (0.0)	0 (0.0)	0 (0.0)	3 (2.6)	18 (15.4)
Iceland	51	7 (13.7)	0 (0.0)	0 (0.0)	0 (0.0)	7 (13.7)	3 (5.9)
Ireland	216	21 (9.7)	0 (0.0)	0 (0.0)	2 (0.9)	23 (10.6)	41 (19.0)
Italy	743	94 (12.7)	1 (0.1)	10 (1.3)	15 (2.0)	112 (15.1)	210 (28.3)
Latvia	125	0 (0.0)	0 (0.0)	0 (0.0)	0 (0.0)	0 (0.0)	7 (5.6)
Lithuania	37	6 (16.2)	0 (0.0)	0 (0.0)	1 (2.7)	7 (18.9)	16 (43.2)
Netherlands	616	49 (8.0)	11 (1.8)	10 (1.6)	5 (0.8)	72 (11.7)	74 (11.8)
Norway	249	0 (0.0)	0 (0.0)	0 (0.0)	0 (0.0)	0 (0.00)	22 (8.8)
Poland	51	6 (11.8)	0 (0.0)	0 (0.0)	0 (0.0)	6 (11.8)	6 (11.8)
Portugal	365	36 (9.9)	2 (0.5)	1 (0.3)	17 (4.7)	49 (13.4)	88 (24.1)
Romania	228	9 (3.9)	0 (0.0)	0 (0.0)	12 (5.3)	19 (8.3)	34 (20.6)
Serbia	37	4 (10.8)	0 (0.0)	0 (0.0)	0 (0.0)	4 (10.8)	3 (8.1)
Slovakia	175	6 (3.4)	0 (0.0)	0 (0.0)	2 (1.1)	8 (4.6)	36 (20.6)
Slovenia	69	18 (26.1)	1 (1.4)	0 (0.0)	1 (1.4)	19 (27.5)	16 (23.2)
Spain	1782	171 (9.6)	8 (0.4)	8 (0.4)	35 (2.0)	211 (11.8)	503 (28.2)
Sweden	306	30 (9.8)	17 (5.6)	0 (0.0)	3 (1.0)	44 (14.4)	53 (17.3)
Switzerland	212	11 (5.2)	1 (0.5)	0 (0.0)	1 (0.5)	12 (5.7)	27 (12.7)
UK	3589	249 (6.9)	187 (5.2)	7 (0.2)	74 (2.1)	492 (13.7)	442 (12.3)
All nations	12170	960 (7.9)	238 (2.0)	55 (0.5)	244 (2.0)	1416 (11.6)	2343 (19.3)

Data are presented in absolute numbers (percentage of patients per nation). Note that multiple types of COM per patient possible. N, number; AWF, arterial wave form analysis; Doppler, Doppler ultrasound; PAC, pulmonary artery catheter; COM, cardiac output monitoring; UK, United Kingdom.
